# Chemotherapy-induced cognitive impairment and its long-term development in patients with breast cancer: results from the observational CICARO study

**DOI:** 10.1093/oncolo/oyae268

**Published:** 2024-10-14

**Authors:** Anna Kerkmann, Christian Schinke, Adam Dordevic, Johannes Kern, Nikola Bangemann, Josefine Finck, Jens-Uwe Blohmer, Klemens Ruprecht, Jens C Göpfert, Carolin Otto, Bianca Materne, Matthias Endres, Wolfgang Boehmerle, Petra Huehnchen

**Affiliations:** Charité—Universitätsmedizin Berlin, Corporate Member of Freie Universität Berlin and Humboldt-Universität zu Berlin, Klinik für Neurologie mit Experimenteller Neurologie, 10117 Berlin, Germany; Charité—Universitätsmedizin Berlin, Corporate Member of Freie Universität Berlin and Humboldt-Universität zu Berlin, Klinik für Neurologie mit Experimenteller Neurologie, 10117 Berlin, Germany; Berlin Institute of Health, Charité—Universitätsmedizin Berlin, 10178 Berlin, Germany; Charité—Universitätsmedizin Berlin, Corporate Member of Freie Universität Berlin and Humboldt-Universität zu Berlin, Klinik für Neurologie mit Experimenteller Neurologie, 10117 Berlin, Germany; Charité—Universitätsmedizin Berlin, Corporate Member of Freie Universität Berlin and Humboldt-Universität zu Berlin, Klinik für Gynäkologie mit Brustzentrum, 10117 Berlin, Germany; Charité—Universitätsmedizin Berlin, Corporate Member of Freie Universität Berlin and Humboldt-Universität zu Berlin, Klinik für Neurologie mit Experimenteller Neurologie, 10117 Berlin, Germany; Carl-Thiem-Klinikum Cottbus, Klinik für Senologie und Systemische Gynäkoonkologie mit Brustzentrum, 03048 Cottbus, Germany; Charité—Universitätsmedizin Berlin, Corporate Member of Freie Universität Berlin and Humboldt-Universität zu Berlin, Klinik für Neurologie mit Experimenteller Neurologie, 10117 Berlin, Germany; Berlin Institute of Health, Charité—Universitätsmedizin Berlin, 10178 Berlin, Germany; Charité—Universitätsmedizin Berlin, Corporate Member of Freie Universität Berlin and Humboldt-Universität zu Berlin, Klinik für Neurologie mit Experimenteller Neurologie, 10117 Berlin, Germany; NMI Natural and Medical Sciences Institute, University of Tübingen, 72770 Reutlingen, Germany; Charité—Universitätsmedizin Berlin, Corporate Member of Freie Universität Berlin and Humboldt-Universität zu Berlin, Klinik für Neurologie mit Experimenteller Neurologie, 10117 Berlin, Germany; Charité—Universitätsmedizin Berlin, Institut für Biometrie und Klinische Epidemiologie, 10117 Berlin, Germany; Charité—Universitätsmedizin Berlin, Corporate Member of Freie Universität Berlin and Humboldt-Universität zu Berlin, Klinik für Neurologie mit Experimenteller Neurologie, 10117 Berlin, Germany; Berlin Institute of Health, Charité—Universitätsmedizin Berlin, 10178 Berlin, Germany; Charité—Universitätsmedizin Berlin, Corporate Member of Freie Universität Berlin and Humboldt-Universität zu Berlin, NeuroCure Cluster of Excellence, 10117 Berlin, Germany; Charité—Universitätsmedizin Berlin, Center for Stroke Research Berlin, 10117 Berlin, Germany; German Center for Neurodegenerative Diseases (DZNE), partner site Berlin, 10117 Berlin, Germany; German Center for Cardiovascular Diseases (DZHK), partner site Berlin, 10117 Berlin, Germany; Charité—Universitätsmedizin Berlin, Corporate Member of Freie Universität Berlin and Humboldt-Universität zu Berlin, Klinik für Neurologie mit Experimenteller Neurologie, 10117 Berlin, Germany; Berlin Institute of Health, Charité—Universitätsmedizin Berlin, 10178 Berlin, Germany; Charité—Universitätsmedizin Berlin, Corporate Member of Freie Universität Berlin and Humboldt-Universität zu Berlin, NeuroCure Cluster of Excellence, 10117 Berlin, Germany; Charité—Universitätsmedizin Berlin, Corporate Member of Freie Universität Berlin and Humboldt-Universität zu Berlin, Klinik für Neurologie mit Experimenteller Neurologie, 10117 Berlin, Germany; Berlin Institute of Health, Charité—Universitätsmedizin Berlin, 10178 Berlin, Germany; Charité—Universitätsmedizin Berlin, Corporate Member of Freie Universität Berlin and Humboldt-Universität zu Berlin, NeuroCure Cluster of Excellence, 10117 Berlin, Germany

**Keywords:** cognitive, impairment, breast cancer, quality of life, neurotoxicity, CICI

## Abstract

**Background:**

Chemotherapy-induced cognitive impairment (CICI) is a well-recognized side effect of breast cancer treatment. However, prospective long-term evaluations of CICI using standardized neuropsychological tests are scarce.

**Patients and Methods:**

This prospective longitudinal cohort study investigated cognitive dysfunction and its impact on quality of life and everyday functioning in patients with breast cancer receiving first-line chemotherapy compared to patients with breast cancer without chemotherapy. Assessment occurred prior to chemotherapy, postchemotherapy (median 6 months), and 2-3 years later. We used standardized neuropsychological tests, questionnaires, and scales to assess patients’ quality of life and functioning. Additionally, serum analysis for neurodegenerative markers and autoantibodies was conducted.

**Results:**

We included *n* = 53 patients. Overall cognitive function declined statistically significantly (*P* = .046) postchemotherapy compared to control patients, mostly driven by a reduced figural memory (*P* = .011). Patients who received chemotherapy showed a greater reduction in quality of life (increased fatigue symptoms, *P* = .023; reduced Karnofsky index, *P* < .001); however, without a statistically significant effect on cognitive decline. The neurodegenerative markers Neurofilament light chain (NfL) and phosphorylated Neurofilament heavy chain (pNfH) increased statistically significantly (*P* < .001) postchemotherapy and pNfH correlated with overall cognitive function. After 2-3 years, both cognitive performance and quality of life were comparable between chemotherapy-treated and control patients.

**Conclusion:**

Our findings suggest that chemotherapy statistically significantly contributes to overall cognitive dysfunction in patients with breast cancer, which disappears after 2-3 years, indicating a recovery in both objectively measurable cognitive function and subjective quality of life. Future research should examine larger sample sizes and explore screening indicators, particularly pNfH.

## Teaser

Chemotherapy-induced cognitive impairment is a well-recognized side effect of breast cancer treatment. This study investigated cognitive dysfunction and its impact on quality of life and everyday functioning in patients with breast cancer receiving first-line chemotherapy compared to patients with breast cancer without chemotherapy.

**Implications for Practice**

Since measurable cognitive impairment occurs in about 1 in 3 patients with locoregional breast cancer undergoing chemotherapy and often affects patients’ quality of life, it is of high clinical importance. Our prospective study suggests that chemotherapy-induced cognitive impairment, which was measurable 2-4 weeks after completion of chemotherapy, recovered after 2-3 years and was then comparable to patients with breast cancer who did not receive chemotherapy. This knowledge helps both in communicating with affected patients and in providing appropriate support to patients.

## Introduction

Patients with breast cancer, the most common cancer in women, now have survival rates of around 90% within the first 5 years, resulting in a large number of long-term survivors.^1^ This has intensified the focus on understanding the adverse effects of cancer therapy and their impact on patients’ quality of life. One common adverse effect is chemotherapy-induced cognitive impairment (CICI), also referred to as “chemobrain.”^2^ CICI can significantly impact patients’ quality of life due to self-perceived changes in cognition. These changes can manifest as episodes of mental confusion, difficulties in recalling words, and the need to exert more mental effort to perform everyday activities. This impact encompasses interactions with family, at work, and other aspects of social life.^3^ Patients typically report symptoms of cognitive impairment around the first month of undergoing chemotherapy, with only a small number of patients describing a deterioration of symptoms following the completion of treatment.^4^ Cognitive domains particularly affected are information processing speed, executive function, as well as verbal and visual learning and memory.^5^

Direct neurotoxic effects, oxidative stress, and a dysregulation of the immune system through the release of cytokines as well as vascular damage have all been discussed as possible pathomechanisms of CICI.^6^ Several radiologic studies underline the existence of chemotherapy-induced changes 1 month after chemotherapy with subsequent partial recovery.^7^ Additionally, the role of specific autoantibodies against neuronal structures and their influence on the probability of the occurrence and course of CICI is under investigation.^8^ Other factors that increase cognitive impairment are psychological factors such as anxiety, stress, and depression.^9^

Evidence suggests that the chemotherapeutic regimes commonly used in breast cancer treatment, containing anthracyclines, taxanes, and alkylating agents are associated with a higher frequency of CICI than other chemotherapeutic regimes.^4^ The meta-analysis of Whittaker et al^3^ found a pooled prevalence of CICI in patients with breast cancer to be as high as 21%-34% when assessed by objective neuropsychological tests. Notably, some of the studies reported prevalences of self-reported cognitive impairment of up to 83%.^3^

Additionally, previous studies indicate conflicting outcomes, with some suggesting sustained cognitive impairment persisting for months or even years after chemotherapy.^3^ However, only limited data exist on the long-term development assessed by objective neuropsychological tests, particularly in individual patient follow-up.

To address these gaps and contribute to improving patient care and quality of life, this study investigates CICI development in locoregional patients with breast cancer. The focus is on cognitive dysfunction observed 2-4 weeks after completion of standard first-line chemotherapy as well as on long-term effects after 2-3 years using standardized neuropsychological tests. Additionally, we aimed to assess the patients’ self-perceived quality of life and cytoskeleton proteins as novel biomarkers.

## Materials and methods

This study, approved by the ethics committee of Charité—Universitätsmedizin in Berlin (EA4/069/14), adheres to STROBE guidelines. All patients provided written informed consent prior to any study procedures.

### Study design and patients

This prospective longitudinal controlled cohort study (previously registered: CICARO; NCT02753036) investigates the effects of chemotherapy on the cognitive function of patients with breast cancer up to 3 years after completion of chemotherapy.

From January 2017 to March 2020, we consecutively screened all patients with breast cancer treated at the Charité Breast Cancer Center for eligibility in the study. Inclusion and exclusion criteria as well as information on a priori done sample size calculation can be found in Supplementary Material. Two groups were recruited and examined: (1) those undergoing chemotherapy consisting of anthracyclines and alkylating agents followed by taxane treatment and (2) those not requiring chemotherapy as controls. The control patients received radiotherapy and/ or hormonal therapy. All patients were treated surgically (breast-conserving surgery or mastectomy).

The participants underwent a baseline examination (V1, before chemotherapy). The subsequent chemotherapy lasted 4-6 months. Two to 4 weeks after completion of chemotherapy, we carried out a first follow-up examination (V2). Control patients were examined approximately 6 months after V1 (V2). An optional third examination was carried out 2-3 years after V2 (V3).

### Outcome measures

#### Neuropsychological evaluation.

We used a standardized battery and protocol of validated neuropsychological tests, each corresponding to specific cognitive domains, to assess the participants’ cognitive function (Table 1).

**Table 1.** Overview of the neuropsychological tests used to assess the various cognitive (sub)domains; and the questionnaires used to assess subjective impairment as well as possible biases in the interpretation of the results.

Neuropsychological test	Indicator	Subindicator
	Cognitive domain	Cognitive subdomain
Verbal Learning and Memory Test	Verbal memory	Verbal learning Verbal retention Verbal recognition
Rey-Osterrieth Complex Figure test	Figural memory	Figural learning Figural retention
Trail Marking Test A and B	Attention	Information processing attention Divided attention
Stroop Color and Word Test Digit span test backwards Semantic verbal fluency test	Executive function	
*Questionnaires*		
Mehrfachwahl-Wortschatz-Intelligenztest A	IQ-score	
European Organisation for Research and Treatment of Cancer	Quality of life	*Global health status* Global health status *Functional scales* Physical functioning Cognitive functioning Social functioning *Symptom scales* Fatigue Pain Insomnia Systemic therapy side effects
Rasch-based Depression Screening	Depression	
Karnofsky performance status scale	Functional impairment	

For verbal memory assessment, we used the verbal learning and memory test. We defined cognitive subdomains of verbal learning, retention, and recognition. We assessed figural memory with the Rey-Osterrieth Complex Figure test (ROCF). We defined the cognitive subdomains of figural learning and retention. To evaluate attention, we used the Trail Marking Test A and B (TMT A and B). We defined the cognitive subdomains of information processing and divided attention. For the assessment of executive function, we used the Stroop Color and Word Test, Digit Span Test Backwards, and Semantic Verbal Fluency Test.

Further information regarding the used tests and the definition of the cognitive subdomains can be found in Supplementary Material.

#### Screening for polyneuropathy.

Previously, we reported the presence of chemotherapy-induced polyneuropathy (CIPN) in the same cohort with additional patients with ovarian cancer.^10^ We performed a standardized neurological examination according to the validated reduced version of the Total Neuropathy Score (TNSr).^11^ Performance and evaluation is detailed in Supplementary Material. We use the previously published data to assess a possible correlation between CIPN and CICI.

#### Questionnaires.

In order to assess subjective impairment and possible biases in the interpretation of our results, we used standardized and validated questionnaires. We assessed the following patient-reported outcomes: IQ score, quality of life, depression, and functional impairment (Table 1).

We determined an approximation of the patients’ IQ scores at V1 using the Mehrfach-Wortschatz-Intelligenztest A, a German multiple-choice vocabulary intelligence test. We assessed different aspects of patient-reported quality of life and cancer- or treatment-associated symptoms with the Quality of Life Questionnaire of the European Organisation for Research and Treatment of Cancer (EORTC-QLQ-C30). In our evaluation, we focused on the function scales and symptom scores (subindicators) of the EORTC-QLQ-C30 listed in Table 1. To screen for depression, we used the Rasch-based Depression Screening (DESC-I). We used the Karnofsky performance status scale (Karnofsky index) to evaluate the patients’ performance status. Details on the structure and evaluation of the questionnaires can be found in Supplementary Material.

#### Serum analysis.

We analyzed the patients’ serum for biomarkers of neuronal or glial injury, ie, determining the concentration of Neurofilament light chain (NfL), phosphorylated neurofilament heavy chain (pNfH), glial fibrillary acidic protein (GFAP), and Tau protein as well as the presence of specific autoantibodies associated with CICI. The autoantibodies examined and the description of the serum analysis procedure can be found in Supplementary Material and Table S1.

### Data processing and statistical analysis

Due to the expected general learning effects in the short time span between the evaluation points V1 and V2, the neuropsychological examinations cannot be considered as independent assessments. To address this, we calculated the change from baseline for each patient-specific value at both follow-up time points (V2−V1 and V3−V1). As changes from baseline in individual tests do not provide much insight into a specific phenotype of cognitive dysfunction, we aimed to group various tests into specific cognitive domains. For this, the individual’s difference from baseline in a specific test was standardized to the mean and SD of the control group by performing z-standardization (control group) and t-standardization (chemotherapy group), respectively.^8^ Then, composite domain scores for each cognitive domain were calculated using the mean of the standardized z-/t-values of all domain subtests. An overall composite cognitive score (total cognitive function) was obtained by averaging all composite domain scores.

For serum analysis, values below the detection limit of the tests were set to the lowest limit of detection by default in order not to further decrease the sample size. Missing data were not imputed.

For the statistical analysis of the differences between the chemotherapy and control group, we used the Mann-Whitney *U* test (*P* ≤ 0.05) due to the small sample size. This also justified our decision not to implement corrections for multiple testing. Further, we explored potential correlations between the total cognitive score and the serum concentrations of the neurodegenerative markers, age, as well as TNSr using a Spearman’s rank correlation. Additionally, a simple linear regression was applied to investigate potential influences of fatigue, pain symptoms (EORTC), or everyday functioning performance (Karnofsky index) on changes in the total cognitive score. For all analysis, Graph Pad Prism version 9 (Graphpad Software, Boston, MA) was used.

## Results

**Figure 1.**
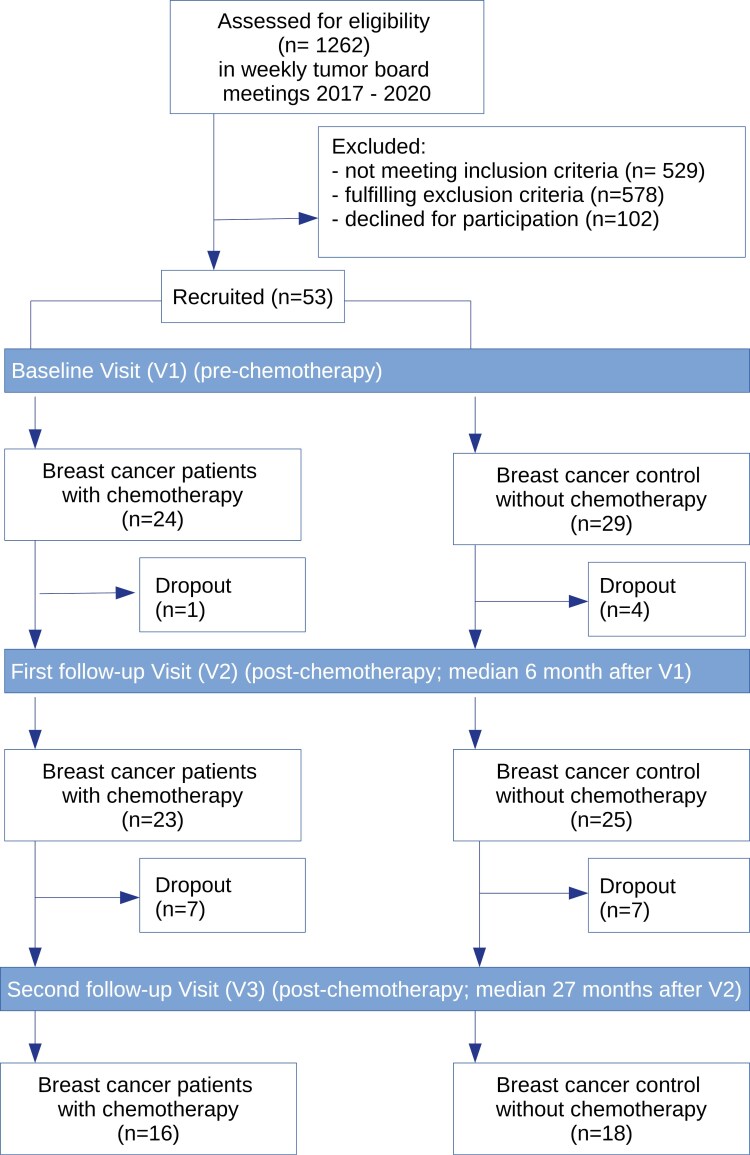
CONSORT diagram. Flow chart of the patients included in the study and their distribution within the study points with representation of dropouts.

The recruitment of patients for the CICARO study was stopped prematurely in March 2020 due to the SARS-CoV-2 pandemic and subsequent local and state restrictions. By this time, we had recruited a total of *n* = 53 patients for the baseline examination V1. For the first follow-up examination V2, 5 patients dropped out, resulting in a total number of *n* = 48. For the optional long-term follow-up V3, we were able to examine *n* = 34 patients. The CONSORT diagram is depicted in Figure 1. Dropout reasons are displayed in Supplementary Material.

The median time interval between V1 and V2 was 6 months in both groups (range in the chemotherapy group: 5-18 months; in the control group: 5-9 months). Between V2 and V3, the median time interval was 27.5 months in the chemotherapy group (range: 25-38 months) and 27 months in the control group (range: 24-39 months).

### Patient characteristics

In our cohort, patients included were exclusively women. Table 2 gives an overview of the patients’ demographics. We note that the age was slightly but significantly lower in the chemotherapy than in the control group (median in chemotherapy group: 50 years [range: 27-61]; median in control group: 54 years [range: 40-68]; *P* = .02). However, Spearman’s rank correlation revealed no significant correlation between the age and total cognitive function. The premorbid IQ values were slightly, but not significantly higher in the chemotherapy group. The patients were quite homogeneous in terms of school education, highest educational degree, and ethnic origin. Patients in the chemotherapy group showed more advanced AJCC stages (American Joint Commission on Cancer) compared to control patients, but these did not correlate with the development of CICI and the degree of recovery. All but one patient undergoing chemotherapy received a combination of epirubicin plus cyclophosphamide followed by (nab-) paclitaxel. Five individuals additionally received carboplatin in conjunction with taxane treatment. One patient received solely docetaxel, instead of paclitaxel, in combination with carboplatin. There were no significant differences between the groups for adjuvant endocrine and Her2/neu-directed therapy.

**Table 2.** Patients’ characteristics

	Chemotherapy group (*n* = 24)	Control group (*n* = 29)	*P*-value
Age, median [range]	50 [27, 61]	54 [40, 68]	**.02**
IQ-score, median [range]	123 [100, 139]	119 [95, 143]	.996
Years of school education, median [range]	13 [10,13]	12 [10,13]	.1
*Highest educational degree, count (share)*			
None	1 (4.5%)	0 (0.0%)	.526
Apprenticeship	4 (18.2%)	9 (34.6%)	
Completed university degree or technical college	17 (77.3%)	17 (65.4%)	
*Ethnic origin, count (share)*			
Central Europe	21 (95.5%)	22 (84.6%)	.28
Mediterranean region	1 (4.5%)	2 (7.7%)	
Asia	0 (0.0%)	2 (7.7%)	
*ER*			
Count (share)	15 (65.2%)	21 (87.5%)	.093
Median in % [range]	80 [0, 100]	100 [0, 100]	**.037**
*PR*			
Count (share)	15 (65.2%)	18 (75%)	.534
Median in % [range]	10 [0, 100]	77.5 [0, 100]	.238
*MIB1*			
Count (share)	22 (100%)	20 (100%)	>.999
Median in % [range]	22.5 [5 80]	1 [2, 20]	**<.001**
*HER2/neu, count (share)*			
No expression	9 (39.1%)	6 (30.0%)	.947
Low expression	9 (39.1%)	12 (60.0%)	
Moderate expression	2 (8.7%)	2 (10.0%)	
High expression	3 (13.0%)	0 (0.0%)	
*AJCC stage, count (share)*			
0	0 (0.0%)	4 (13.8%)	**0.001**
IA	3 (12.5%)	15 (51.7%)	
IB	3 (12.5%)	0 (0.0%)	
IIA	8 (33.3%)	6 (20.7%)	
IIB	5 (20.8%)	2 (6.9%)	
IIIA	1 (4.2%)	1 (3.4%)	
IIIB	3 (12.5%)	0 (0.0%)	
IIIC	1 (4.2%)	0 (0.0%)	
*Additional adjuvant endocrine therapy, count (share)*			
None	11 (47.8%)	6 (24.0%)	0.314
Tamoxifen	4 (17.4%)	11 (44.0%)	
Letrozol	2 (8.7%)	8 (32.0%)	
Trastuzumab + pertuzumab	3 (13.0 %)	0 (0.0%)	
Others	3 (13.0%)	2 (8.0%)	
*Additional Her2/neu-directed therapy, count (share)*			
Not received	9 (39.1%)	6 (30.0%)	0.51
Received	14 (60.9%)	14 (70.0%)	

Additional adjuvant endocrine therapy in the category “Others” included: denosumab (1 patient), ribociclip (1 patient in combination with tamoxifen), atezolizumab (1 patient) in the chemotherapy group; and anastrozol (2 patients) in the control group.

Statistically significant difference between the chemotherapy and the control group is displayed in bold.

Missing values: 1 patient value missing for IQ-score, ER, PR, MIB1, and HER2/neu and 2 patients for years of school education in chemotherapy group and 4 in the control group.

Abbreviations: AJCC: American Joint Commission on Cancer; ER, estrogen receptor positivity; HER2/neu: human epidermal growth factor receptor 2 positivity; *n*, number of patients evaluated; MIB1, antibody directed at the protein Ki-67 (ER, PR, and MIB1 positivity ranges from 1% to 100% of positive tumor nuclei); PR, progesterone receptor positivity.

### Neuropsychological evaluation

**Figure 2.**
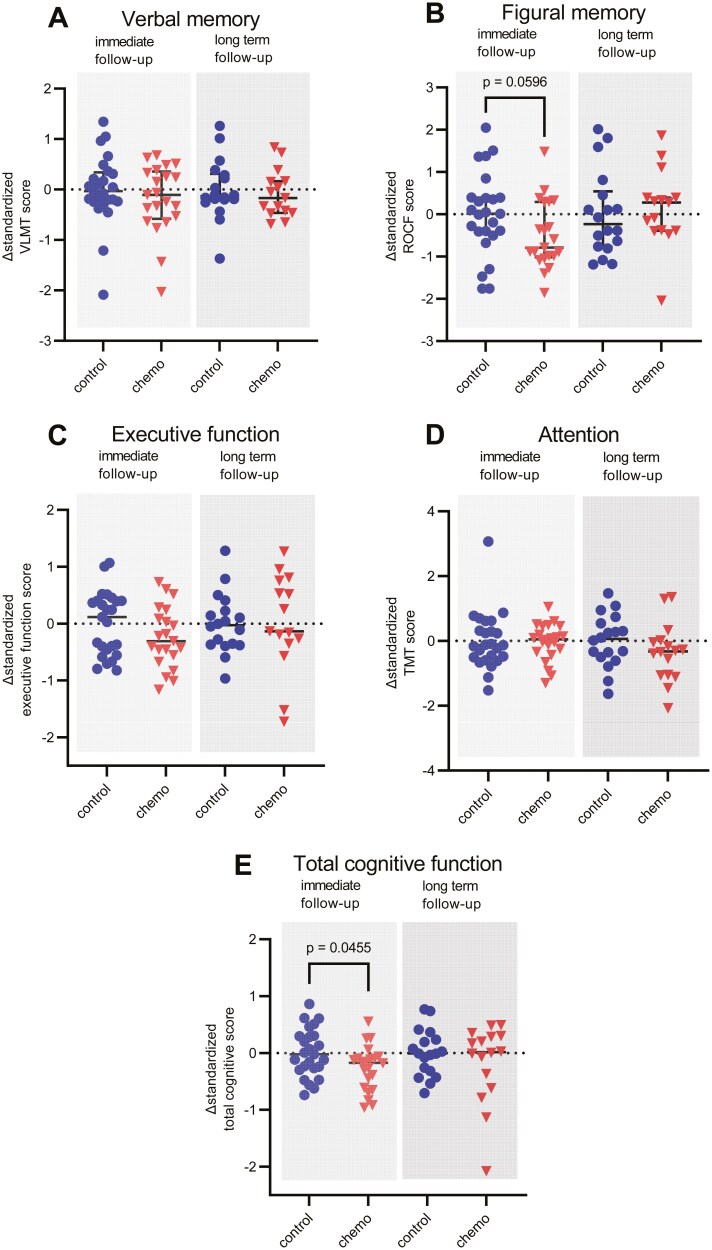
Results of the cognitive domains of the neuropsychological assessment. The results of verbal memory (A), figural memory (B), executive function (C), and attention (D), as well as a total cognitive score (E) are presented as individual change from baseline, both for the first follow-up 2-4 weeks after completion of chemotherapy (V2−V1) and the long-term follow-up after 2-3 years (V3−V1). The medians and the interquartile ranges are presented for each domain for the chemotherapy and the control groups. If there is a statistically significant difference between the groups or a *P*-value close to significance, these are displayed. Abbreviations: ROCF: Rey-Osterrieth Complex Figure Test; TMT: Trail Marking Test; VLMT: Verbal Learning and Memory Test.

First, we were interested in the effects of chemotherapy treatment on cognitive function 2-4 weeks after completion of chemotherapy (V2). In our cohort, we observed a trend toward a reduced performance in figural and verbal memory, as well as in executive function, when compared to baseline and the control group (Supplementary Table S2). In the individual tests, however, only the differences in figural retention reached statistical significance (median in chemotherapy group: 1 point [IQR: −0.5, 6.25]; median in control group: 6 points [IQR: 2.75, 8.25]; *P* = .011; Supplementary Figure S1E). Furthermore, we found a significant difference in the total cognitive function (*P* = .046; Figure 2E). Although this result is mainly driven by the differences in figural memory, the differences in the figural memory domain score itself did not reach statistical significance (median of the chemotherapy group: −0.79 points [IQR: −1.01, 0.29]; control group: 0.01 points [IQR: −0.46, 0.4]; *P* = .06). With the exception of attention, all domain scores showed a tendency toward reduced cognitive function in the chemotherapy compared to the control group (Figure 2; Supplementary Table S2). Other additional treatments such as endocrine or Her2/neu-directed therapies did not correlate with the overall cognitive function.

The second part of the study examined how these changes in cognitive function developed after a period of 2-3 years (V3). In our cohort, total cognitive function was comparable between the groups (median in chemotherapy group: 0.01 points [IQR: −0.56, 0.29]; median in control group: −0.01 points [IQR: −0.34, 0.27]; *P* = .878). All the other domain scores also showed no clear tendency (Figure 2). The same development could be seen for the figural retention test score (median in chemotherapy group: 2 points [IQR: 0, 3.5]; median in control group: 0.75 points; [IQR: −2, 5.12]; *P* = .549). Only the Stroop Test, an assessment of executive function, showed a deterioration compared to baseline and the control group (median in chemotherapy group: −9 points [IQR: −13.75, 4.75]; median in control group: 0.5 points [IQR: −6, 11.5], *P* = .029; Supplementary Table S2 and Figure S1G).

In our cohort, around 61% of chemotherapy-treated patients also developed symptoms of a clinically relevant CIPN, as previously reported.^10^ We conducted Spearman’s rank correlation analysis to investigate the relationship between CIPN severity and decreased performance in cognitive testing, revealing no significant correlation between CIPN and cognitive function (Spearman *r* = −0.11 [95% CI, −0.39 to 0.19]; *P* = .472).

### Patient-reported quality of life and symptoms

**Figure 3.**
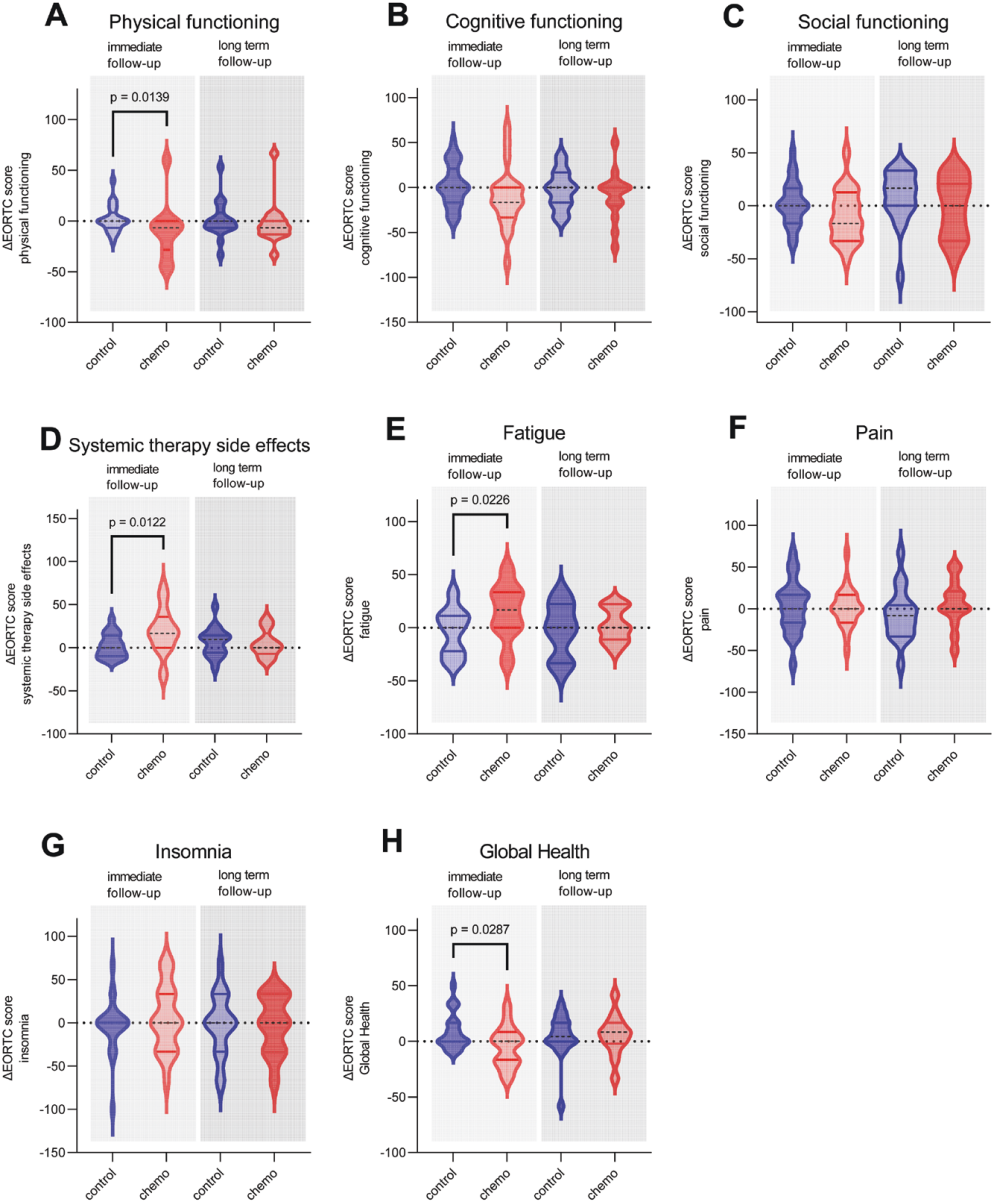
Test results of the quality of life questionnaire of the European Organisation for Research and Treatment of Cancer (EORTC-QLQ-BR23). The results of physical functioning (A), cognitive functioning (B), social functioning (C), systemic therapy side effects (D), fatigue (E), pain (F), insomnia (G), and Global Health (E) are presented as individual change from baseline, both for the first follow-up 2-4 weeks after completion of chemotherapy (V2−V1) and the long-term follow-up after 2-3 years (V3−V1). The medians and the interquartile ranges are presented for each scale for the chemotherapy and the control groups. If there is a statistically significant difference between the groups or a *P*-value close to significance, these are displayed.

Next, we assessed the patient’s quality of life and daily functioning. Here, we first examined the changes 2-4 weeks after completion of chemotherapy (V2) compared to baseline. In our cohort, the results of EORTC-QLQ-C30 and BR23 showed a deterioration of all functional scales in the chemotherapy group, but only physical functioning decreased statistically significantly compared to the controls (median in chemotherapy group: −6.67 AU [IQR: −28.33, 0]; median in control group: 0 AU [IQR: −6.67, 8.33]; *P* = .0139; Figure 3A-C; Supplementary Table S3). Further, we found a significant increase in fatigue symptoms (median in chemotherapy group: 16.67 AU [IQR: 0, 33.33]; median in control group: 0 AU [IQR: −22.22, 11.11]; *P* = .023) and systemic therapy side effects (median in chemotherapy group: 16.67 AU [IQR: 0, 35.71]; median in control group: 0 AU [IQR: −9.58, 14.29]; *P* = .012). The medians of the other symptom scores did not differ between the baseline and the control group (Figure 3D-G). However, chemotherapy-treated patients rated their overall global health status score significantly lower (median in chemotherapy group: 0 AU [IQR: −16.67, 8.33]; median in control group: 0 AU [IQR: 0, 16.67]; *P* = .029; Figure 3H). Further, the Karnofsky index, evaluating performance in activities of daily living, was significantly lower in the chemotherapy than the control group (median in chemotherapy group: −10 % [IQR: −10, 0]; median in control group: 0 % [IQR: 0, 0]; *P* < .001; Supplementary Table S4). Given these results, we were interested whether fatigue or functional impairment and additionally pain had an impact on our neuropsychological test results. We performed a linear regression, which revealed no significant influences of these factors on the total cognitive function score, with low *R*^*2*^ values between 0.01 and 0.02. With regards to additional treatments other than chemotherapy, we only observed a slight correlation between additional endocrine therapy and social functioning after the end of chemotherapy, respectively after 6 months in control patients (*P* = .048, Spearman *r* = −0.318), whereas Her2/neu-directed treatments were not correlated with changes in quality of life.

In the next step, we wanted to examine how these patient-reported symptoms and effects on quality of life changed after a period of 2-3 years (V3). We observed no difference to baseline in any of the symptom scores, including fatigue and the systemic therapy side effect scores, and overall comparability to the control group (Figure 3D-G). The physical functioning scale showed a tendency of deterioration, which, however, did not reach statistical significance (median in chemotherapy group: −6.667 AU [IQR: −13.33, 0]; median in control group: 0 AU (IQR: [−6.67, 8.33]; *P* = .272; Figure 3A). The global health status also showed no significant difference between the groups (Figure 3H; Supplementary Table S3). Further, the Karnofsky index deteriorated since the initial examination, but to the same extent as in the control group (Supplementary Table S4).

To rule out a possible influence of depressive symptoms on cognitive function, we carried out a depression screening (DESC score). At baseline (V1), one patient in the chemotherapy group and 2 patients in the control group had values that indicated a potential depressive episode. Two to 4 weeks after completion of chemotherapy (V2), the patients’ median score did not differ compared to baseline and the control group. After 2-3 years (V3), the chemotherapy group showed no difference in the median score compared to baseline, whereas the control group showed a slightly higher median (no statistically significant difference between the groups; Supplementary Table S4).

### Autoantibodies and neurodegenerative markers

Lastly, we were interested in the presence of specific autoantibodies associated with the occurrence of cognitive decline in patients with cancer. The percentages of autoantibody seropositivity detected did not differ significantly between the groups (Supplementary Material). The frequency of NMDA antibodies in our cohort, as the most common autoantibodies, was 8.7% for NMDA-IgA, 8.7% for NMDA-IgM, and 0% for NMDA-IgG in the chemotherapy group, and 16.7% for NMDA-IgA, 4.17% for NMDA-IgM, and 4.17% for NMDA-IgG in the control group (no statistically significant difference between the groups).

**Figure 4.**
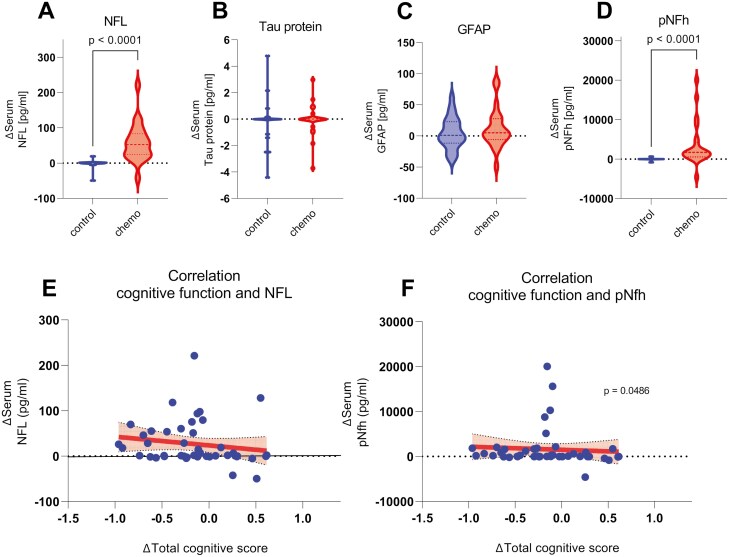
Serum concentrations of the neurodegenerative markers and their correlation with total cognitive function. For the neurodegenerative markers NfL, tau protein, GFAP, and pNfH, the serum concentrations (in pg/mL) including the medians and interquartile ranges for the chemotherapy and control groups are presented as individual changes from the first follow-up 2-4 weeks after completion of chemotherapy compared to baseline (V2−V1). In addition, the correlation analysis between the change in serum concentrations of NfL and pNfH and total cognitive function are presented. If there is a significant difference between the groups/a significant correlation or a *P*-value close to statistical significance, these are displayed. Abbreviations: GFAP, glial fibrillary acidic protein; NfL, Neurofilament light chain; pNfH, phosphorylated neurofilament heavy chain.

Further, we examined the concentrations of neurodegenerative markers in the serum. Patients showed a significantly greater increase in the concentrations of NfL (median in chemotherapy group: 52.45 pg/mL [IQR: 24.2, 83.05]; median in control group: −0.1 pg/mL (IQR: [−1.43, 1.12]; *P* < .001) and pNfH (median in chemotherapy group: 1708 pg/mL [IQR: 556.7, 4270]; median in control group: −5.78 pg/mL [IQR: −37.33, 10.99]; *P* < .001) 2-4 weeks after completion of chemotherapy treatment than control patients. The median concentrations of Tau protein and GFAP did not differ significantly between the groups and over time (Figure 4A-D; Supplementary Table S5). To evaluate whether the serum concentrations of these markers correlated to cognitive impairment, we conducted a Spearman’s rank correlation analysis. In our cohort, we found a weak but significant correlation between the decline of total cognitive function and the serum concentration of pNfH (Spearman *r* = −0.3 [95% CI, −0.55 to 0.01]; *P* = .049). However, when comparing the pretreatment serum levels of chemotherapy patients who later experienced CICI and those who did not, we found no differences in pNfH concentrations (Supplementary Material). The serum concentrations of NfL, tau protein, and GFAP showed no significant correlation with cognitive function (Figure 4E, F). Additionally, pNfh as well as NfL did not show a significant correlation with figural retention as the most impaired subdomain.

## Discussion

### Summary of the main results

In this prospective longitudinal controlled cohort study on patients with breast cancer, we investigated CICI and patients’ self-perceived quality of life over a period of up to 3 years. We found that total cognitive function and particularly figural retention as part of the figural memory were significantly lower in patients 2-4 weeks after completion of chemotherapy compared to controls who did not receive chemotherapy. However, these differences disappeared within 2-3 years. Further, we observed a significant reduction in quality of life and daily functioning in patients 2-4 weeks after completion of chemotherapy compared to controls (ie, EORTC-QLQ-C30 questionnaire: higher fatigue symptoms and systemic therapy side effects, lower overall global health status and physical functioning; lower Karnofsky index). However, these differences also disappeared within 2-3 years. Serum concentrations of pNfH mildly but significantly correlated with cognitive impairment, while significantly increased NfL serum levels did not.

### Interpretation of the results and comparison to other studies

Our results clearly indicate that chemotherapy can lead to cognitive impairment in patients with breast cancer, particularly in figural memory, which can be objectively measured 2-4 weeks after completion of chemotherapy. This aligns with previous findings showing that CICI is a common side effect in patients with breast cancer^.4,12-15^ While our study also hinted at declines in verbal memory and executive function, these differences were not statistically significant, which might be due to the overall low sample size of our cohort. Interestingly, in their meta-analyses, Jansen et al^5^ also found that only visual memory was significantly impaired among chemotherapy-treated patients across all comparison types.^.^ Further, animal studies support visuospatial deficits as a most pronounced symptom of CICI after paclitaxel chemotherapy.^16^ In part, this is substantiated by radiological studies in patients with cancer identifying a decrease in hippocampal and parahippocampal volume.^7,12,17^ Additionally, inhibition of adult hippocampal neurogenesis has been observed in animal models.^16^ Nevertheless, in the even bigger meta-analyses of Ono et al,^13^ visuospatial function was not significantly impaired, and they found the greatest limitations postchemotherapy in processing speed and executive function. Ibrahim et al^14^ also found the biggest limitation in executive function, and additionally attention. The discrepancy between the results might be due to different sample sizes, but warrants further investigations.^13^

Our results suggest that the cognitive function of patients with breast cancer recovers after chemotherapy within 2-3 years, returning to a comparable level to patients with cancer who have not received chemotherapy. The only notable difference in long-term follow-up was found in the Stroop Test, a component of executive function. However, as other subtests assessing executive function and the domain score remained unchanged, we are cautious in the interpretation of this result. Our results are concordant with previous results. Previous meta-analyses even found patients treated with chemotherapy performed better in some follow-up examinations than at baseline, possibly due to higher motivation, learning effects, or other sources of bias.^12,13^

Further, our results imply a greater limitation in patients’ overall quality of life 2-4 weeks after completion of chemotherapy compared to controls. Previous studies support this observation.^3,6^ We also found a slight correlation between endocrine therapy and social functioning, which may have skewed our results in this aspect. However, there was no significant difference in the use of additional endocrine or Her2/neu-directed therapy between the groups, suggesting this to be an independent effect and not linked to systemic chemotherapy. Our long-term results imply that the quality of life of chemotherapy recipients is similar to that of nonrecipients after 2-3 years. However, the physical function scale continued to show a tendency toward deterioration, although without reaching statistical significance. This could be partly explained by the high prevalence of CIPN even 2-3 years after completion of chemotherapy in our cohort, which is also consistent with other studies.^18^

While a large number of our chemotherapy-treated patients developed symptoms of CIPN, which is concordant with previous literature_,_^19^ we did not observe a significant relationship between CIPN severity and decreased performance in cognitive testing. This implies that patients with CIPN are not more likely to suffer from CICI and vice versa, but may also indicate that the pathomechanisms differ between peripheral and central nervous system toxicity.

Further, we explored serum neurodegenerative markers as potential novel biomarkers for the early diagnosis of CICI. While we could previously link increased NfL serum levels with peripheral nervous system toxicity from paclitaxel treatment, which correlated with CIPN severity in our cohort,^10^ our findings also showed an increase of pNfH, which—to a lesser extent—also significantly correlated with cognitive decline. Natori et al^20^ also evaluated pNfH and observed dose-dependent increases in pNfH serum levels in patients with breast cancer undergoing chemotherapy_._ However, they found no significant difference in the neuropsychological test results between patients with and without elevated pNfH values. Yet, the neuropsychological results of their study may not be sensitive enough, as they used self-administered tests instead of standardized test batteries.

In our study, typical structural markers only found in the CNS like Tau protein or GFAP remained consistent and therefore did not reflect CNS toxicity. In their meta-analysis, Schroyen et al^21^ also found no increase in Tau protein serum levels in patients undergoing chemotherapy.

Nevertheless, we only measured increased pNfH serum levels when the cognitive damage was already present. There were no differences in pNfH serum levels prior to treatment when comparing the subjects who later experienced CICI and those who did not, which clearly limits pNfH’s use as a predictive marker. To assess the usefulness of pNfH as a potential diagnostic biomarker, more regular evaluation before, during, and after chemotherapy combined with objective neuropsychological testing should be done in the future.

### Strengths and limitation

Although about one in 3 locoregional patients with breast cancer who undergo chemotherapy show measurable cognitive impairment, few studies explored its long-term trajectory with standardized tests.^3^ Our study’s primary strength lies in the comprehensive individual assessment of cognitive function over a period of up to 3 years, from the initial baseline examination before chemotherapy to the final follow-up examination. Additionally, we used alternating versions of the standardized neuropsychological tests at the different examination points to minimize practice effects, as generally recommended_._^12^ Furthermore, our study stands out by using a cancer control group instead of healthy controls, as most previous studies did. Previous studies have shown an overall decline in cognitive performance in patients with cancer compared to healthy, age-matched individuals, suggesting that some of the changes observed were due to the cancer itself.^3,22^Further, poorer effect sizes have been found when using healthy comparison groups.^13^ Additionally, our patients received highly comparable chemotherapy regimens in terms of substances, dose, and frequency, which also increases the value of the study. Due to strict inclusion and exclusion criteria, our groups were more homogeneous than in previous studies and we were able to exclude most potential biases of cognitive dysfunction from our cohort. In earlier longitudinal studies, the great heterogeneity was often considered problematic.^12^ Further, we investigated several factors potentially influencing cognitive function and biases: First, we were able to negate a possible influence on our results from fatigue, pain, and everyday functioning performance. Second, a depression screening revealed no significant differences between the groups regardless of the examination points. Third, we assessed the premorbid IQ scores and found no correlation with cognitive function. Finally, we screened for autoantibodies associated with cognitive impairment and found that the overall incidence of NMDA antibodies, as the most common autoantibodies, and others to be comparable between the groups and to that of the (healthy) general population.^23^

The biggest limitation of our study is the low sample size, which was due to the necessary premature termination of recruitment during the SARS-CoV-2 pandemic. In addition, our study only included women, thus these results might be partly driven by gender effects. Although the patients are quite homogeneous in terms of schooling, highest educational degree, and ethnic origin, most included patients are of Central European origin, which represents only a subgroup of potentially affected individuals. While none of the included patients received any medication targeted to treat their cognitive dysfunction or fatigue symptoms, all patients were encouraged to undergo psychological consultation and therapy as part of their routine cancer treatment. Thereby, we cannot rule out a possible influence of these on our results. Furthermore, due to the lack of data on additional radiation treatment, we cannot determine its influence on quality of life, while we consider its impact on cognitive dysfunction to be minimal due to the specific localized radiation treatment of the breast and not the brain.

## Conclusion

Our study stands as one of the few to investigate CICI and its impact on patients’ quality of life over a period of several years using standardized neuropsychological tests. Our findings reveal that CICI, notably impairment in figural memory, and a decline in quality of life are objectively measurable 2-4 weeks after completion of chemotherapy in patients with locoregional breast cancer. Cognitive impairment might correlate with serum concentrations of pNfH. However, after a period of 2-3 years, the changes in cognition and the perception of quality of life disappeared. The results, therefore, imply that the decline in cognitive function and the subjectively reduced quality of life recover in the longer term. They are then again comparable with patients with breast cancer who have not received chemotherapy.

Further studies using standardized neuropsychological tests and including a larger number of patients are required to detect more subtle differences and to obtain more precise results on long-term prevalences. Moreover, further studies are needed on the early diagnosis of CICI, in particular, on a possible correlation with neurodegenerative markers, especially pNfH.

## Supplementary material

Supplementary material is available at *The Oncologist* online.

oyae268_suppl_Supplemental_Material

## Data Availability

The data underlying this article are available in Zenodo, at https://dx.doi.org/10.5281/zenodo.11203535.
