# Supplementary material for: Chemotherapy-induced cognitive impairment and its long-term development in patients with breast cancer: results from the observational CICARO study
Source: Oncologist. 2024 Oct 14;30(2):oyae268. doi: 10.1093/oncolo/oyae268 (PMC11881063; doi:10.1093/oncolo/oyae268)
Supplement: oyae268_suppl_Supplemental_Material [file oyae268_suppl_supplemental_material.docx]

**Supplement**

**Supplemental material & methods**

*Inclusion and exclusion criteria to participate in the study*

To participate, patients had to meet the following inclusion criteria: first-time diagnosis of a loco-regional mamma-carcinoma, aged 18-70 years, Karnofsky-Index ≥70% and at least eight years of school education. Exclusion criteria included: documented postoperative delirium, previously diagnosed mild cognitive impairment or dementia, current moderate and/or severe depressive episode, previous or current alcohol or drug abuse, anaemia with haemoglobin levels ≤8g/dl or haemoglobin loss of ≥2g/dl between evaluation points, or history of previous neurotoxic chemotherapy treatment.

*Description of a priori sample size calculations*

We conducted a priori sample size calculation using a standardized total cognitive score as the primary endpoint. Anticipating a moderate effect size based on literature suggesting an effect of -0.4 to -0.6 from chemotherapy on cognitive function^1^, we used an expected effect size of -0.5. For the sample size calculation, a paired-samples t-test was used as this provides a more conservative approach. With an α-error of 0.05 and an adjusted statistical power of 0.8, a minimum of n=34 patients per group was calculated. To account for potential dropouts and ensure robustness, we aimed to recruit n=40 patients per group.

*Evaluation of the verbal memory with the Verbal Learning and Memory Test (VLMT)*

The Verbal Learning and Memory Test (VLMT) includes learning and recalling a 15-word list across five trials, followed by a single presentation and immediate recall of a 15-word distractor list. Participants are then asked to recall the items after the distraction, as well as after a retention interval of 20-30 minutes. Additionally, a delayed yes-no recognition task is conducted. Performance was measured by the total items recalled during learning trials (verbal learning score: sum of VLMT trials 1 to 5), decline in delayed recall compared to the last trial (verbal retention score: difference of VLMT trial 7 and 5), and correct hits adjusted for false positives in a recognition task (verbal recognition score). To mitigate learning effects between visits, we used two different test versions. The VLMT is recognized as a valid measure of verbal episodic declarative memory^2^.

*Evaluation of the figural memory using the Rey-Osterrieth Complex Figure test (ROCF)*

The Rey-Osterrieth Complex Figure test (ROCF) involves copying a complex geometric figure by hand, followed by a first free recall after three minutes and second delayed recall 30 minutes later. We utilized Osterrieth's standardized scoring system, breaking down the figure into 18 components and assigning points (0 to 2) for accuracy and position. The maximum score is 36 points^3^. We divide the figural memory performance in a figural learning performance score (score of the first recall) and a figural retention performance score (score of the second delayed recall). The ROCF is widely employed for evaluating visuospatial constructional ability and non-verbal memory^4^.

*Evaluation of the attention using the Trail Marking Test A and B (TMT A and B)*

TMT A involves connecting 25 encircled numbers in numerical order, while TMT B requires connecting 25 encircled numbers and letters in alternating numerical and alphabetical order. Performance is measured in the time needed to complete each part of the test. We divide the attention performance into information processing attention (TMT A) and divided attention (TMT B). The TMT is recognized for measuring cognitive domains such as processing speed and visual-motor skills^5^.

*Evaluation of executive function using the Stroop Color and Word Test, Digit Span Test Backwards, and Semantic Verbal Fluency Test*

The Stroop Color and Word Test presents a list of colour words to the examinee, where the ink colour does not correspond to the word's meaning. The task is to quickly name the ink colour, while disregarding the word's semantic meaning. Performance is assessed by measuring the time required to complete the task and uncorrected errors. The Stroop Test gains valuable insights into executive functions such as attentional processes and inhibitory control^6,7^. The Digit Span Test Backwards requires memorizing a series of digits in reverse order. The test starts with a length of two digits and progressively increases to a maximum of seven digits. There are always two different attempts within a digit length. The test continues until the participant provides two incorrect responses within a digit length or reaches the predetermined maximum list size. Performance is scored based on correct sequences (maximum 12 points). In the Semantic Verbal Fluency Test, patients list words from a specific category within one minute, with the score based on the number of words correctly formed. To minimize learning effects, two different categories were used at different evaluation points. The Semantic Verbal Fluency Test is widely used to assess executive function^8^.

*Screening for polyneuropathy*

We utilized the validated Total Neuropathy Score in the reduced version (TNSr) to evaluate the presence of chemotherapy-induced polyneuropathy^9^. For this purpose, we assessed pin sensibility, vibration sensibility with a Riedel-Seyfert tuning fork, muscle strength according to Medical Research Council (MRC) and reflex status, each of which were graded regarding their severity of impairment. We conducted an abbreviated protocol of nerve conduction studies in these patients assessing compound motor action potential and motor conduction velocity of the peroneal nerve as well as sensory nerve action potential and sensory conduction velocity of the sural nerve according to the TNSr grading system using a standard ENG/EMG device (Schreiber & Tholen, Germany).

*Questionnaires used to assess subjective impairment and possible biases in the interpretation of the results*

The Quality Of Life Questionnaire of the European Organisation for Research and Treatment of Cancer (EORTC-QLQ-C30) is a 30-question-core questionnaire, which incorporates nine multi-item scales: five functional scales (physical, role, cognitive, emotional, and social); three symptom scales (fatigue, pain, as well as nausea and vomiting); and a global health and quality of life scale. Additionally, several single-item symptom measures are included. The EORTC breast cancer module adds 23 questions including the functional scales of body image, sexual functioning, sexual enjoyment and future perspective as well as the symptom scales of (systemic) breast cancer therapy side effects. We deployed the official EORTC scoring manual to calculate raw scores of each scale, which are then transferred into standardized linear scores ranging from 0 to 100^10^. Studies indicate that the questionnaire is a valid and reliable tool to assess the quality of life of cancer patients^11^.

The Rasch-based Depression Screening (DESC-I) consists of 10 questions with multiple choice options. There is an established cut-off score of ≥12 points to classify a patient as possibly depressed. It has been proven to be a valid tool for depression screening^12^.
The Karnofsky performance status scale uses a 11-point scale to grade cancer patients overall health and everyday functioning ranging from 100% (no evidence of disease, no symptoms) to 0% (death).

*Serum Analysis*

We analysed the patients’ serum for concentrations of neurodegenerative markers and presence of specific autoantibodies. For this purpose, we withdrew 2 x 5ml whole blood, which we centrifuged at 2000 g for 10 min and then snap froze the supernatant and stored it at -80°C until analysation. Serum concentrations of the neurodegenerative markers neurofilament light chain (NfL), phosphorylated neurofilament heavy chain (pNFh), glial fibrillary acidic protein (GFAP) and Tau protein was measured with Single Molecule Array (SIMOA) from samples obtained at V1 and V2. Measurements were done at the Natural and Medical Sciences Institute (NMI) at the University of Tübingen using commercially available kits and following the manufacturer’s instructions.

Furthermore, we screened for the presence of specific autoantibodies associated with cancer and cognitive impairment in serum samples obtained pre-chemotherapy treatment at V1 (Supplementary Table ST1). We did not screen for autoantibodies at the follow-up time points as chemotherapy treatment typically does not induce antibody production. The samples were analysed using commercially available assays at EUROIMMUN Medizinische Labordiagnostika AG (Germany), which included cell-based assays and immunohistochemistry on frozen brain tissues (specifically, rat hippocampus, rat cerebellum, and monkey cerebellum). Indirect immunofluorescence was carried out using the BIOCHIP mosaics method. To validate the results, additional immunoblot tests using EUROLINE was conducted. In cases where autoantibodies were identified through immunohistochemistry but yielded negative results in cell-based assays and EUROLINE, they were categorized as autoantibodies with reactivity against unknown epitopes.

**Supplemental results**

*Reasons for patients dropping out of the study*

Reasons for patients dropping out of the study included the restart of chemotherapeutic treatment (two patients), death (one patient), time constraints (four patients), family reasons (two patients), relocation (two patient), the unwillingness to continue participation (four patients) and issues related to reachability (four patients).

*Autoantibodies associated with cognitive impairment*

Our findings revealed that three patients (12.5%) within the chemotherapy group exhibited the presence of antibodies against NMDA-IgA and IgM (two patients) and Flotillin (one patient). In comparison, five patients within the control group (17.9%) showed positive results for the following antibodies: NMDA-IgA (two patients), NMDA-IgA and IgM (one patient), NMDA-IgA and IgG (one patient), and ARHGAP26 (one patient).

*pNfH serum levels*

To evaluate the potential usefulness of pNfH as a biomarker, we additionally compared the prior-treatment serum levels of subjects who experienced CICI with those who did not. For this, we defined chemotherapy-treated patients as cognitively impaired if their total cognitive score was lower than the median of the control group, and compared their serum levels with those of the other patients who received chemotherapy. There was no significant difference.

**Supplemental figures and tables**

**Supplementary Table S1: Autoantibodies associated with cancer and cognitive impairment investigated in V1.**

| IgG autoantibodies | α-amino-3-hydroxy-5-methyl-4-isoxazolepropionic acid receptor 1/2, amphiphysin, aquaporin 4, RhoGTPase-activating protein 26, ATP1A3, carbonic anhydrase related proteins VIII, contactin-associated protein-like 2, collapsin response-mediator protein 5, dipeptidyl-peptidase-like protein 6, Flotillin1/2, gamma-aminobutyric-acid A receptor, gamma-aminobutyric-acid B receptor, glutamic acid decarboxy lase 65, glial fibrillary acidic protein, glutamate receptor delta 2, glycine receptor, Homer protein homolog 3, Hu, immunoglobulin LON5, inositol 1,4,5-trisphosphate receptor 1, leucine-rich glioma-inactivated 1, Ma2, metabotropic glutamate receptor 1, metabotropic glutamate receptor 5, myelin oligodendrocyte glycoprotein, myelin, anti-neuroendothelium, neurexin, neurochondrin, N-methyl-D-aspartate receptor, recoverin, Ri, septin complex, Tr, Yo, and zinc finger 4 |
| --- | --- |
| IgA and IgM autoantibodies | N-Methyl-D-Aspartat (NMDA) |

**Supplementary Table S2: Detailed test results of the neuropsychological evaluation.**

| *Test results are presented as individual change from baseline, both for the first follow-up examination two to four weeks after completion of chemotherapy and respectively after approximately six months after the baseline examination in the control group (V2-V1); and for the long-term follow-up after two to three years (V3-V1). If there is a significant difference between the chemotherapy and the control group these are displayed in bold. Abbreviations: n: number of patients evaluated, IQR: interquartile range* | | | | | | | | | | | |
| --- | --- | --- | --- | --- | --- | --- | --- | --- | --- | --- | --- |
| **First follow-up examination** | | | | | | | | | | | |
| Domain | Sub-domain | Chemotherapy group | | | | Control group | | | | | p-value |
|  |  | n | Median | Range | IQR | n | Median | Range | IQR |  | |
| Figural memory | Figural learning  (in points) | 21 | 2 | [-4, 11.5] | [1, 7] | 25 | 5 | [-7, 16] | [2.5, 7.5] | .1413 | |
|  | Figural retention  (in points) | 21 | 1 | [-4, 14.5] | [-0.5, 6.25] | 25 | 6 | [-3.5, 16] | [2.75, 8.25] | **.0106** | |
|  | **Total score** | 21 | -0,7897 | [-1.86, 1.478] | [-1.013, 0.2902] | 25 | 0,01185 | [-1.757, 2.049] | [-0.4551, 0.3953] | .0596 | |
| Verbal memory | Verbal learning  (in number of words) | 21 | -1 | [-19, 13] | [-3, 6] | 25 | 3 | [-18, 17] | [-1.5, 9] | .1572 | |
|  | Verbal retention  (in number of words) | 21 | -1 | [-4, 2] | [-1, 0.5] | 25 | 0 | [-5, 2] | [-2, 1] | .9778 | |
|  | Verbal recognition  (in number of words) | 22 | 0 | [-3, 4] | [0, 1] | 25 | 0 | [-2, 2] | [0, 1] | .9149 | |
|  | **Total score** | 21 | -0.1051 | [-2.033, 0.676] | [-0.58, 0.3581] | 25 | -0.0327 | [-2.083, 1.342] | [-0.2395, 0.3381] | .4213 | |
| Attention | Information processing attention  (in seconds) | 22 | -2 | [-30, 11] | [-6.25, 3.52] | 25 | -3 | [-15, 45] | [-6, 2] | .6759 | |
|  | Divided attention  (in seconds) | 22 | -3 | [-58, 24] | [-10.24, 1] | 25 | -11 | [-40, 27] | [21, 4] | .4949 | |
|  | **Total score** | 22 | 0.05211 | [-1.307, 1.05] | [-0.3602, 0.4493] | 25 | -0.1269 | [-1.52, 3.072] | [-0.551, 0.4639] | .7756 | |
| Executive function | a) Stroop-test (in seconds) | 22 | -4.5 | [-65.96, 17] | [-13.5, 3.5] | 25 | -5 | [-22, 33] | [-10, 3] | .5643 | |
|  | b) Word fluency test  (in number of words) | 21 | 4 | [-6, 18] | [-1.5, 7.5] | 25 | 5 | [-2, 17] | [0.5, 8] | .3712 | |
|  | c) Digit span test backwards  (in number of correct digit series) | 22 | 0 | [-3, 2] | [-1, 1] | 25 | 0 | [-4, 3] | [-1, 0.5] | .8082 | |
|  | **Total score** | 21 | -0.308 | [-1.164, 0.7308] | [-0.6138, 0.1645] | 25 | 0.1156 | [-0.821, 1.069] | [-0.5051, 0.401] | .1803 | |
| **Total cognitive function** | **Total cognitive score** | 22 | -0.1750 | [-0.9615, 0.5498] | [-0.6225, 0.08658] | 25 | -0.02471 | [-0.739, 0.861] | [-0.2804, 0.2995] | **.0455** | |
| **Second long-term follow-up examination** | | | | | | | | | | | |
| Figural memory | Figural learning  (in points) | 15 | 4 | [-9, 11] | [0, 5] | 18 | 0.25 | [-8, 12] | [-1.625, 4.5] | .2607 | |
|  | Figural retention  (in points) | 15 | 2 | [-7, 10.5] | [0, 3.5] | 18 | 0.75 | [-2.5, 11] | [-2, 5.125] | .549 | |
|  | **Total score** | 15 | 0.2756 | [-2.041, 1.861] | [-0.3876, 0.3928] | 18 | -0.2303 | [-1.187, 2.012] | [-0.7725, 0.5416] | .3564 | |
| Verbal memory | Verbal learning  (in number of words) | 15 | -1 | [-6, 22] | [-2, 10] | 18 | 2 | [-13, 12] | [-4.75, 8.5] | .6743 | |
|  | Verbal retention  (in number of words) | 15 | 0 | [-2, 2] | [0, 1] | 18 | 1 | [-1, 7] | [0, 2] | .2395 | |
|  | Verbal recognition  (in number of words) | 16 | 0 | [-1, 6] | [0, 0] | 18 | 0 | [-2, 2] | [-1, 0.25] | .868 | |
|  | **Total score** | 15 | -0.167 | [-0.6857, 0.8374] | [-0.4633, 0.1638] | 18 | -0.1348 | [-1.368, 1.258] | [-0.2134, 0.309] | .4915 | |
| Attention | Information processing attention  (in seconds) | 16 | -5.5 | [-39, 17] | [-13.25, 0.25] | 18 | -1 | [-14, 30] | [-7.75, 6] | .1832 | |
|  | Divided attention  (in seconds) | 16 | 4 | [-74, 54] | [-8.25, 18.25] | 18 | 11.5 | [-42, 33] | [-14.75, 19.25] | .6637 | |
|  | **Total score** | 16 | -0.3221 | [-2.076, 1.352] | [-1.06, 0.001414] | 18 | 0.06131 | [-1.628, 1.473] | [-0.5252, 0.5955] | .2373 | |
| Executive function | a) Stroop-test (in seconds) | 16 | -9 | [-55.96, 15] | [-13.75, 4.75] | 18 | 0.5 | [-16, 36] | [-6, 11.5] | **.0289** | |
|  | b) Word fluency test  (in number of words) | 15 | 3 | [-13, 14] | [-4, 7] | 18 | 1 | [-9, 8] | [-2.25, 3] | .5488 | |
|  | c) Digit span test backwards  (in number of correct digit series) | 16 | 0 | [-3, 5] | [-1, 1.75] | 18 | -1 | [-3, 2] | [-2, 1] | .2644 | |
|  | **Total score** | 15 | -0.1351 | [-1.724, 1.266] | [-0.353, 0.7552] | 18 | -0.0266 | [-0.9647, 1.281] | [-0.3698, 0.2709] | .63 | |
| **Total cognitive function** | **Total cognitive score** | 16 | 0.0147 | [-2.076, 0.492] | [-0.5574, 0.2944] | 18 | -0.0095 | [-0.7038, 0.767] | [-0.3439, 0.2689] | .8782 | |


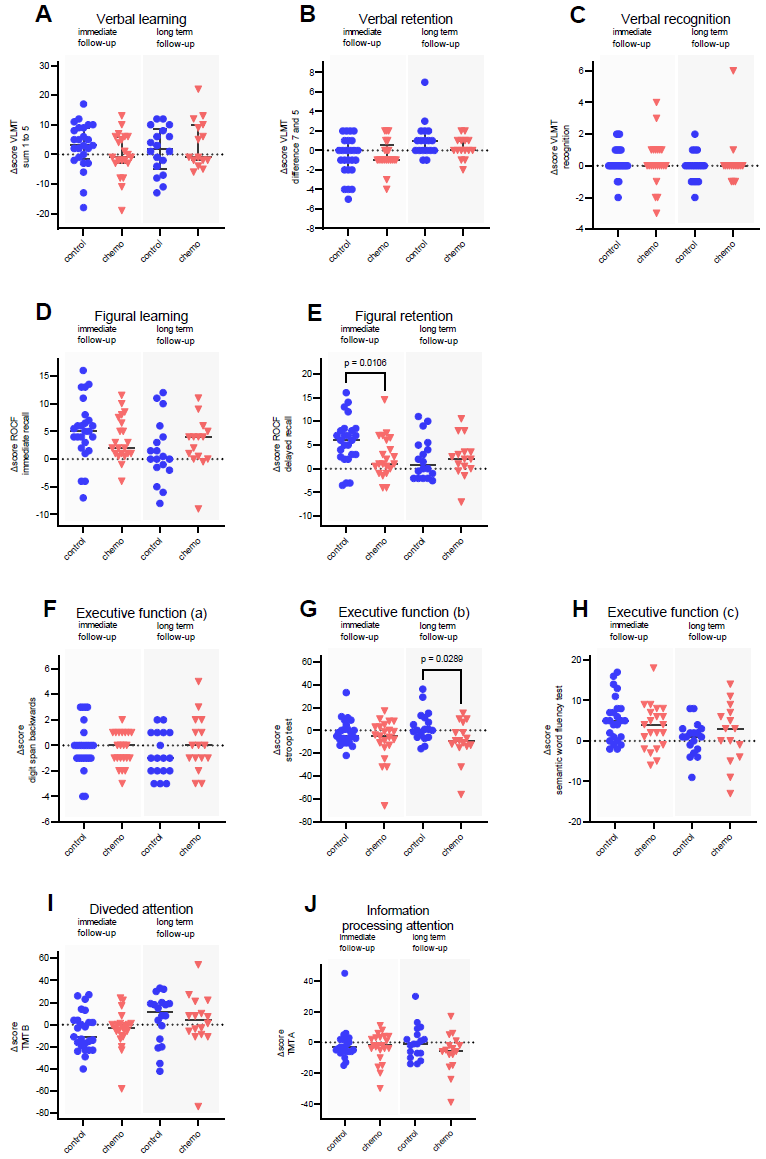


**Supplementary Figure S1: Single test results of the neuropsychological assessment.** The results of verbal learning (A), verbal retention (B), verbal recognition (C), figural learning (D), figural retention (E), executive function (F-H), divided attention (I), and information processing attention (J) are presented as individual change from baseline, both for the first follow-up examination two to four weeks after completion of chemotherapy and respectively after approximately six months after the baseline examination in the control group (V2-V1); and for the long-term follow-up after two to three years (V3-V1). The medians and the interquartile ranges are presented for each test for the chemotherapy and the control group. If there is a significant difference between the groups or a p-value close to significance, these are displayed.

Abbreviations: VLMT: Verbal Learning and Memory Test, ROCF: Rey-Osterrieth Complex Figure Test; TMT: Trail Marking Test

ALT-TEXT: Graphs on the observed changes in cognitive function (single test results of the neuropsychological evaluation) with subfigures from A to J, illustrating changes at the immediate and late follow-up in control vs. chemotherapy groups.

**Supplementary Table S3: Detailed test results of the EORTC-QLQ-BR23.**

| *Test results are presented as individual change from baseline, both for the first follow-up examination two to four weeks after completion of chemotherapy and respectively after approximately six months after the baseline examination in the control group (V2-V1); and for the long-term follow-up after two to three years (V3-V1). If there is a significant difference between the chemotherapy and the control group these are displayed in bold. Abbreviations: EORTC-QLQ-BR23: Quality of Life Questionnaire of the European Organisation for Research and Treatment of Cancer; n: number of patients evaluated, IQR: interquartile range* | | | | | | | | | | | | |
| --- | --- | --- | --- | --- | --- | --- | --- | --- | --- | --- | --- | --- |
| **First follow-up examination** | | | | | | | | | | | | |
| Scales and scores | | Chemotherapy group | | | | | Control group | | | | | p-value |
|  |  | n | Median | Range | IQR | n | | Median | Range | IQR |  | |
| Functional scales | Physical functioning | 22 | -6.667 | [-46.67, 60] | [-28.33, 0] | 22 | | 0 | [-20, 40] | [-6.667, 8.333] | **.0139** | |
|  | Cognitive functioning | 21 | -16.67 | [-83.33, 66.67] | [-33.33, 0] | 22 | | 0 | [-33.33, 50] | [-16.67, 20.83] | .0961 | |
|  | Social functioning | 20 | -16.67 | [-50, 50] | [-33.33, 12.5] | 21 | | 0 | [-33.33, 50] | [-16.67, 16.67] | .1522 | |
| Symptom scales | Fatigue | 21 | 16.67 | [-33.33, 55.56] | [0, 33.33] | 22 | | 0 | [-33.33, 33.33] | [-22.22, 11.11] | **.0226** | |
|  | Pain | 21 | 0 | [-50, 66.67] | [-16.67, 16.67] | 22 | | 0 | [-66.67, 66.67] | [-16.67, 16.67] | .6118 | |
|  | Insomnia | 20 | 0 | [-66.67, 66.67] | [-33.33, 33.33] | 21 | | 0 | [-100, 66.67] | [0, 0] | .6357 | |
|  | Systemic therapy side effects | 20 | 16.67 | [-33.33, 71.43] | [0, 35.71] | 22 | | 0 | [-14.29, 33.33] | [-9.58, 14.29] | **.0122** | |
| Global health status / QoL | Global health status/QoL | 21 | 0 | [-33.33, 33.33] | [-16.67, 8.333] | 22 | | 0 | [-8.333, 50] | [0, 16.67] | **.0287** | |
| **Second long-term follow-up examination** | | | | | | | | | | | | |
| Functional scales | Physical functioning | 15 | -6.667 | [-33.33, 66.67] | [-13.33, 0] | 18 | | 0 | [-33.33, 53.33] | [-6.667, 8.333] | .2722 | |
|  | Cognitive functioning | 14 | 0 | [-66.67, 50] | [-20.83, 0] | 18 | | 0 | [-33.33, 33.33] | [-16.67, 16.67] | .5567 | |
|  | Social functioning | 14 | 0 | [-50, 33.33] | [-33.33, 20.83] | 18 | | 16.67 | [-66.67, 33.33] | [0, 33.33] | .3154 | |
| Symptom scales | Fatigue | 14 | 0 | [-22.22, 22.22] | [-11.11, 22.22] | 18 | | 0 | [-44.44, 33.33] | [-33.33, 22.22] | .684 | |
|  | Pain | 14 | 0 | [-50, 50] | [-4.167, 20.83] | 18 | | -8.333 | [-66.67, 66.67] | [-33.33, 4.167] | .1475 | |
|  | Insomnia | 12 | 0 | [-66.67, 33.33] | [-33.33, 33.33] | 18 | | 0 | [-66.67, 66.67] | [-33.33, 33.33] | .9192 | |
|  | Systemic therapy side effects | 13 | 0 | [-15.08, 33.33] | [-7.143, 16.67] | 18 | | 9.524 | [-23.81, 47.62] | [-5.952, 14.29] | .6422 | |
| Global health status / QoL | Global health status/QoL | 14 | 8.333 | [-33.33, 41.67] | [-2.083, 16.67] | 18 | | 4.167 | [-58.33, 33.33] | [0, 16.67] | .9169 | |

**Supplementary Table S4: Detailed results of the Karnofsky-Index and DESC-score.**

| *Test results are presented as individual change from baseline, both for the first follow-up examination two to four weeks after completion of chemotherapy and respectively after approximately six months after the baseline examination in the control group (V2-V1); and for the long-term follow-up after two to three years (V3-V1). If there is a significant difference between the chemotherapy and the control group these are displayed in bold.*  *Abbreviations: DESC-score: Rasch-based Depression Screening, n: number of patients evaluated, IQR: interquartile range* | | | | | | | | | |
| --- | --- | --- | --- | --- | --- | --- | --- | --- | --- |
| **First follow-up examination** | | | | | | | | | |
|  | Chemotherapy group | | | | Control group | | | | p-value |
|  | n | Median | Range | IQR | n | Median | Range | IQR |  |
| *Karnofsky-Index* | 23 | -10 | [-20, 0] | [-10, 0] | 24 | 0 | [-10, 10] | [0, 0] | **<.0001** |
| DESC-score | 20 | 0 | [-811] | [-1,1] | 25 | 0 | [-5,19] | [-1, 5] | .2584 |
| **Second long-term follow-up examination** | | | | | | | | | |
| *Karnofsky-Index* | 16 | -10 | [-20, 10] | [-17.5, 0] | 18 | -10 | [-10, 10] | [-10, 0] | .31 |
| DESC-score | 13 | 0 | [-14, 8] | [-2, 2] | 18 | 1 | [-9,37] | [-0.25, 7] | .2684 |

**Supplementary Table S5: Detailed results of the serum concentrations of the neurodegenerative markers.**

| *Test results are presented as individual change from baseline, both for the first follow-up examination two to four weeks after completion of chemotherapy and respectively after approximately six months after the baseline examination in the control group (V2-V1); and for the long-term follow-up after two to three years (V3-V1). If there is a significant difference between the chemotherapy and the control group these are displayed in bold.*  *Abbreviations: NFL: neurofilament light chain, pNfh: phosphorylated neurofilament heavy chain, GFAP: glial fibrillary acidic protein, n: number of patients evaluated, IQR: interquartile range* | | | | | | | | | |
| --- | --- | --- | --- | --- | --- | --- | --- | --- | --- |
| **First follow-up examination** | | | | | | | | | |
|  | Chemotherapy group | | | | Control group | | | | p-value |
|  | n | Median | Range | IQR | n | Median | Range | IQR |  |
| *NFL[pg/ml]* | 22 | 52.45 | [-42.2, 221] | [24.2, 83.05] | 24 | -0.1 | [-49.47, 19.14] | [-1.428, 1.115] | **<.0001** |
| pNfh *[pg/ml]* | 22 | 1708 | [-4597, 20062] | [556.7, 4270] | 24 | -5.783 | [-822.9, 626.4] | [-37.33, 10.99] | **<.0001** |
| Tau protein *[pg/ml]* | 22 | 0 | [-3.717, 2.975] | [-0.2025, 0.1811] | 24 | 0 | [-4.42, 4.78] | [-0.0525, 0.0525] | .8821 |
| GFAP *[pg/ml]* | 22 | 5.134 | [-48.72, 87.83] | [-5.725, 27.84] | 24 | 0.9233 | [-37.29, 62.09] | [-11.48, 22.92] | .4132 |

**References:**

1. Jansen CE, Miaskowski C, Dodd M, Dowling G, Kramer J. A metaanalysis of studies of the effects of cancer chemotherapy on various domains of cognitive function. *Cancer*. 2005;104(10):2222-2233. doi:10.1002/cncr.21469

2. Helmstaedter C, Durwen HF. VLMT: Verbaler Lern- und Merkfähigkeitstest: Ein praktikables und differenziertes Instrumentarium zur Prüfung der verbalen Gedächtnisleistungen [VLMT: A useful tool to assess and differentiate verbal memory performance]. In: *Schweizer Archiv Für Neurologie, Neurochirurgie Und Psychiatrie*. ; 1990:141(1), 21-30.

3. Osterrieth PA. Le test de copie d’une figure complexe. In: *Archives de Psychologie*. ; 1944:30, 206-356.

4. Shin MS, Park SY, Park SR, Seol SH, Kwon JS. Clinical and empirical applications of the Rey–Osterrieth Complex Figure Test. *Nat Protoc*. 2006;1(2):892-899. doi:10.1038/nprot.2006.115

5. Bowie CR, Harvey PD. Administration and interpretation of the Trail Making Test. *Nat Protoc*. 2006;1(5):2277-2281. doi:10.1038/nprot.2006.390

6. Periáñez JA, Lubrini G, García-Gutiérrez A, Ríos-Lago M. Construct Validity of the Stroop Color-Word Test: Influence of Speed of Visual Search, Verbal Fluency, Working Memory, Cognitive Flexibility, and Conflict Monitoring. *Archives of Clinical Neuropsychology*. 2021;36(1):99-111. doi:10.1093/arclin/acaa034

7. Scarpina F, Tagini S. The Stroop Color and Word Test. *Front Psychol*. 2017;8. doi:10.3389/fpsyg.2017.00557

8. Quaranta D, Piccininni C, Caprara A, Malandrino A, Gainotti G, Marra C. Semantic Relations in a Categorical Verbal Fluency Test: An Exploratory Investigation in Mild Cognitive Impairment. *Front Psychol*. 2019;10:2797. doi:10.3389/fpsyg.2019.02797

9. Cavaletti G, Frigeni B, Lanzani F, et al. The Total Neuropathy Score as an assessment tool for grading the course of chemotherapy‐induced peripheral neurotoxicity: comparison with the National Cancer Institute‐Common Toxicity Scale. *J Peripheral Nervous Sys*. 2007;12(3):210-215. doi:10.1111/j.1529-8027.2007.00141.x

10. Fayers PM, Aaronson N, Bjordal K, Groenvold M, Curran D, Bottomley A. *EORTC QLQ-C30 Scoring Manual: This Manual Is Intended to Assist Users with Scoring Procedures for the QLQ-C30 Version 3 and Earlier, and the QLQ Supplementary Modules*. 3rd ed. EORTC; 2001.

11. Salas M, Mordin M, Castro C, Islam Z, Tu N, Hackshaw MD. Health-related quality of life in women with breast cancer: a review of measures. *BMC Cancer*. 2022;22(1):66. doi:10.1186/s12885-021-09157-w

12. Forkmann T, Boecker M, Wirtz M, et al. Validation of the Rasch-based Depression Screening in a large scale German general population sample. *Health Qual Life Outcomes*. 2010;8(1):105. doi:10.1186/1477-7525-8-105
